# Preparation of “Cauliflower-Like” ZnO Micron-Sized Particles

**DOI:** 10.3390/ma6115234

**Published:** 2013-11-14

**Authors:** Tamar Gordon, Judith Grinblat, Shlomo Margel

**Affiliations:** Department of Chemistry, The Institute of Nanotechnology and Advanced Materials, Bar Ilan University, Ramat Gan 52900, Israel; E-Mails: gordont1@mail.biu.ac.il (T.G.); Judith.Grinblat@biu.ac.il (J.G.)

**Keywords:** ZnO micrometer-sized particles, PDVB microspheres, fluorescence

## Abstract

Porous polydivinyl benzene (PDVB) microspheres of narrow size distribution were formed by a single-step swelling process of template uniform polystyrene microspheres with divinyl benzene (DVB), followed by polymerization of the DVB within the swollen template microspheres. The PDVB porous particles were then formed by dissolution of the template polystyrene polymer. Unique “cauliflower-like” ZnO microparticles were prepared by the entrapping of the ZnO precursor ZnCl_2_ in the PDVB porous microspheres under vacuum, followed by calcination of the obtained ZnCl_2_-PDVB microspheres in an air atmosphere. The morphology, crystallinity and fluorescence properties of those ZnO microparticles were characterized. This “cauliflower-like” shape ZnO particles is in contrast to a previous study demonstrated the preparation of spherical shaped porous ZnO and C-ZnO microparticles by a similar method, using zinc acetate (ZnAc) as a precursor. Two diverted synthesis mechanisms for those two different ZnO microparticles structures are proposed, based on studies of the distribution of each of the ZnO precursors within the PDVB microspheres.

## 1. Introduction

The directed shaping of materials is a field of high interest for different applications as well as for fundamental questions regarding anisotropic crystallization. Zinc oxide (ZnO) specifically is of great interest in the scientific community due to its wide variety of properties and the applications derived from them, including semiconductor properties, piezoelectricity and UV absorbance [1]. The unique optical properties of ZnO are determined by the size and the morphology of its particles [2,3,4,5]. This was the main motivation for researchers to prepare ZnO particles with different structures and shapes. ZnO particles with various structures were synthesized by different methods, such as chemical vapor deposition [4,6], electrodeposition [7], and hydrothermal methods [2,5,8,9,10,11]. Another approach enabling the preparation of “tailor made” particles is the use of organic polymer template. This approach involves the insertion of ZnO precursor into polymeric template particles, decomposition of the precursor within the template particles to obtain ZnO particles with controlled structure, predetermined by the template structure, and then removal of the template by dissolution or by thermal decomposition. Some examples for ZnO particle synthesis using this approach are the preparation of hollow ZnO microparticles by calcination of hydrozincite coated poly(styrene) beads [12], or poly(methyl methacrylate) impregnated by Zn(NO_3_)_2_ [13], and the synthesis of hexagonal shaped ZnO microparticles, nanowires and nanocubes by employing different “molecular building blocks” to direct the assembly of ordered metal-organic coordination-polymer templates [3,14,15].

Our previous study described the formation of porous spherical shaped micrometer-sized ZnO and C-ZnO particles of narrow sized distribution by entrapping zinc acetate within the pores of uniform polydivinyl benzene (PDVB) porous microspheres. ZnO and C-ZnO microparticles were then prepared by decomposition of these zinc acetate entrapped PDVB particles in an air or inert atmosphere, respectively [16]. In the present study, we demonstrate the preparation of “cauliflower-like” ZnO microparticles, using the same PDVB porous template microspheres. ZnCl_2_ was used as a precursor, and was entrapped within the PDVB template microspheres pores. The “cauliflower-like” ZnO microparticles were then prepared by thermal decomposition of the PDVB template microspheres, in the same manner as the ZnO porous spherical shaped microparticles. However, the different Zn precursors dictated different micro-environments that led to porous sphere ZnO microparticles in one case and “cauliflower-like” ZnO microparticles in the other, as will be discussed.

## 2. Experimental Section

The following chemicals were purchased from Aldrich, Rehovot, Israel, and were used without further purification: zinc chloride (ZnCl_2_, 98%), benzoyl peroxide (BP, 98%), Tetrahydrofuran (THF), dimethylformamide (DMF), divinylbenzene (DVB, 80%), sodium dodecyl sulfate (SDS), polyvinylpyrrolidone (PVP, *M_w_* = 360,000 g/mol), ethanol (HPLC), 2-methoxy ethanol (HPLC), dibutyl phthalate (DBP) and methylene chloride (HPLC). Styrene (S, 99%) was passed through activated alumina (ICN) to remove inhibitors before use. Water was purified by passing deionized water through an Elgastat Spectrum reverse osmosis system (Elga Ltd., High Wycombe, UK). 

Uniform micrometer-sized PDVB microspheres of 5.4 ± 0.1 μm were prepared by a single-step swelling process at room temperature of polystyrene (PS) template microspheres with DBP (swelling solvent) containing DVB and BP, followed by polymerization at elevated temperature and dissolving the PS template part, as described previously [16,17,18]. Briefly, PS template microspheres of 2.3 ± 0.1 μm were swollen up to 8.1 ± 0.2 μm by the following procedure: 1.5 mL DBP containing 10 mg BP and 1.5 mL DVB were added to a 20 mL vial containing 10 mL SDS aqueous solution (0.75% *w*/*v*). Emulsion droplets of the swelling solvent were then formed by sonication (Sonics and Materials, model VCX-750, Ti-horn 20 kHz) of the mixture for 1 min. An aqueous dispersion (3.5 mL) of the PS template microspheres (7% *w*/*v*) was then added to the stirred DBP emulsion. After the swelling of the PS particles was complete and the mixture did not contain any small droplets of the emulsified swelling solvent, as verified by optical microscopy, the monomer within the swollen particles was polymerized by raising the temperature of the shaken vial containing the swollen particles to 73 °C for 24 h. The composite PS/PDVB microspheres produced were then washed from undesired reagents by extensive centrifugation cycles with water and ethanol. Uniform porous crosslinked micrometer-sized PDVB particles of 5.4 ± 0.1 μm were then prepared by dissolving the PS template part of the PS/PDVB composite microspheres with methylene chloride, acetone and DMF. Briefly, PS/PDVB composite particles were dispersed in 50 mL methylene chloride:acetone (1:2), and then shaken at room temperature for *ca.* 15 min. The dispersed microspheres were then centrifuged, and the supernatant containing the dissolved PS template polymer was discarded. This procedure was repeated four times with methylene chloride:acetone (1:2), DMF, ethanol, and water. The obtained crosslinked PDVB microspheres were then dried by lyophilization.

ZnCl_2_-PDVB composite microspheres were prepared by evacuating 100 mg of the PDVB porous template microspheres for 30 min, using a diaphragm vacuum pump in a 50 mL double neck round bottom flask closed with a septum. Then 0.1 mL of ZnCl_2_ solution in THF (20% *w*/*v*) was injected into the round-bottom flask. The PDVB microspheres containing the ZnCl_2_ were then mixed and dried by vacuum evaporation.

“Cauliflower-like” ZnO microparticles were prepared by calcinating the ZnCl_2_-PDVB microspheres in an air atmosphere at 600 °C (wire wound tube furnace TF-25-250/H, Carbolite, Hope, UK).

Optical and fluorescence microscopy images were obtained using an Olympus optical/fluorescent microscope (model AX70, filter WU: excitation 330–385 nm, emission >420 nm). Surface morphology was characterized with a scanning electron microscope (SEM, model Inspect S, FEI, Eindhoven, The Netherland). The samples were coated with gold in vacuum prior to scanning with SEM.

The samples for the TEM analysis were prepared by milling a small amount of the “cauliflower-like” ZnO microparticles, dissolving them in ethanol and dispersing a small drop of the sonnicated solution on a TEM copper grid. Cross-sections of the ZnO-PDVB microspheres were prepared by embedding the particles in a mixture of Pelco^®^ epoxy resin part A and Pelco^®^ slow curing epoxy hardener part B (3:1), sectioned with a microtome (Powertom TG122, Boeckeler, Tucson, AZ, USA) and coated with a thin layer of carbon.

For imaging two transmission electron microscopes were used: 120 kV Technai T12 BIO TWIN and the 200 kV JEOL-JEM2100 LaB_6._ Identification and elemental content studies were performed in TEM or HRTEM mode, using conventional selected area electron diffraction (SAED) technique or Fourier transform analysis of the high resolution images. The characterization and identification of the particles is supported by elemental analysis techniques such as: Energy dispersive X-ray (point EDS) and Line scans, performed in scanning transmission electron mode (STEM).

Brunauer–Emmet–Teller (BET) surface area of the various particles was analyzed by using nitrogen adsorption apparatus (27 Gemini III model 2375, Micrometrics). Prior to the nitrogen adsorption measurements, the samples were degassed at 120 °C for 1 h. Pore-size distributions were determined from the nitrogen desorption isotherms using the Barrett–Joyner–Halenda (BJH) method. Pore volume was estimated from the desorbed amount of N_2_ at *P*/*P*_0_ = 0.9952. Thermal behavior of the particles was measured by thermogravimetric analysis (TGA) and differential scanning calorimetry (DSC), under N_2_ atmosphere. (TC15 system equipped with TGA, model TG-50, and DSC, model DSC-30, Mettler Toledo, Switzerland).

## 3. Results and Discussion

**Figure 1:**
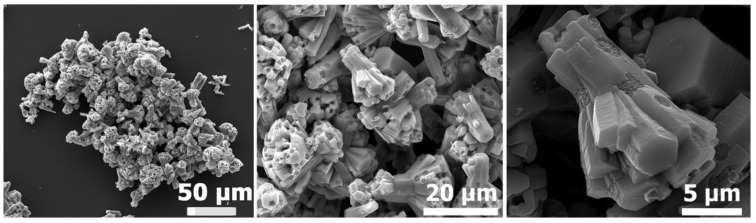
“Cauliflower-like” ZnO microparticles: Scanning electron microscope (SEM) images of different magnifications.

Typical “cauliflower-like” ZnO microparticles, prepared by the addition of ZnCl_2_ to PDVB template microspheres under vacuum, followed by calcination of the obtained ZnCl_2_-PDVB composite microspheres in an air atmosphere, are presented in Figure 1.

**Figure 2:**
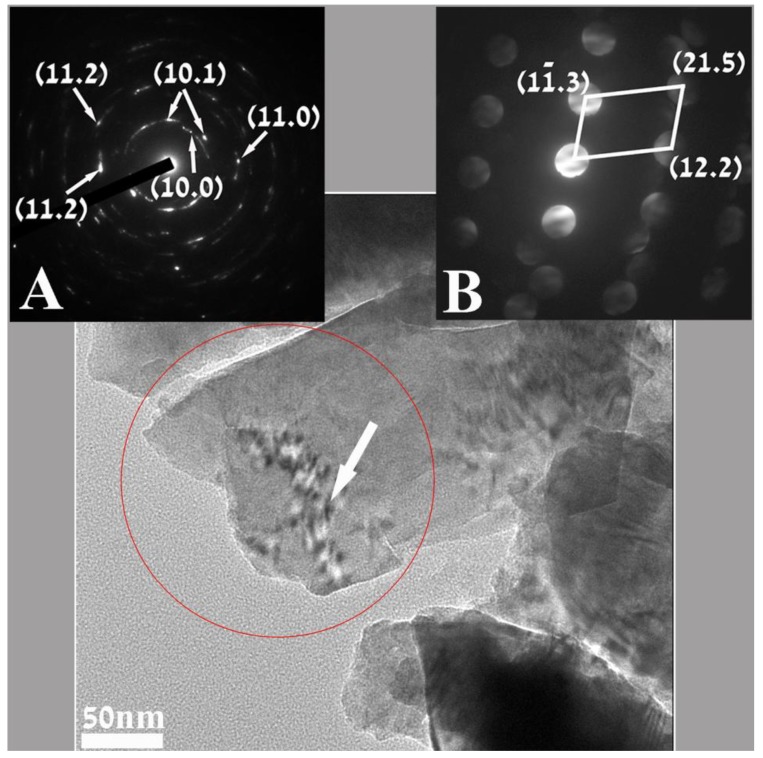
Transmission electron microscopy (TEM) image of representative nanoparticles of milled “Cauliflower-like” ZnO microparticles. Inset (**A**) is the selected area electron diffraction (SAED) taken from a 200 nm area marked by the red cycle, displaying reflections matching the ring diffraction pattern for the hexagonal ZnO. The nano beam electron diffraction (NBD), inset (**B**) recorded from a 4nm area, marked by the arrow, points also to the hexagonal ZnO.

For all transmission electron microscopy (TEM) observation these “Cauliflower-like” ZnO microparticles were milled to smaller sized particles. Figure 2 is a representative TEM micrograph where a few nanoparticles can be discerned in a sample of milled “Cauliflower-like” ZnO microparticles. Both electron diffractions (insets A and B) show reflections that were found to belong to the hexagonal structure of ZnO (unit cell *a* = 3.25 Å; *c* = 5.21 Å pdf file # 01-0899-1397) Inset A is the selected area electron diffraction (SAED) taken from an area of 200 nm, displaying mainly a ring diffraction pattern. The reflections that appear in the SAED can be assigned to the {10.0}, {10.1}, {11.0} and {11.2} family of planes (in the hexagonal structure describing ZnO. The measured distances for these planes are 0.27 nm, 0.24 nm, 0.16 nm and 0.13 nm respectively. The reflection that appears in the nano beam electron diffraction (NBD, inset B) taken from a 4 nm area, as marked by the white arrow, shows clear sets of patterns.

Analysis of one such pattern, (inset B) revealed sets of reflections that could be referred and indexed to the above mentioned structure of ZnO. The distances measured for the reflections were 0.16 nm, 0.1 nm and 0.078 nm matching, respectively, the interplanar spacings *d*_10.3_, *d*_12.2_ and *d*_21.5_ family of planes.

**Figure 3:**
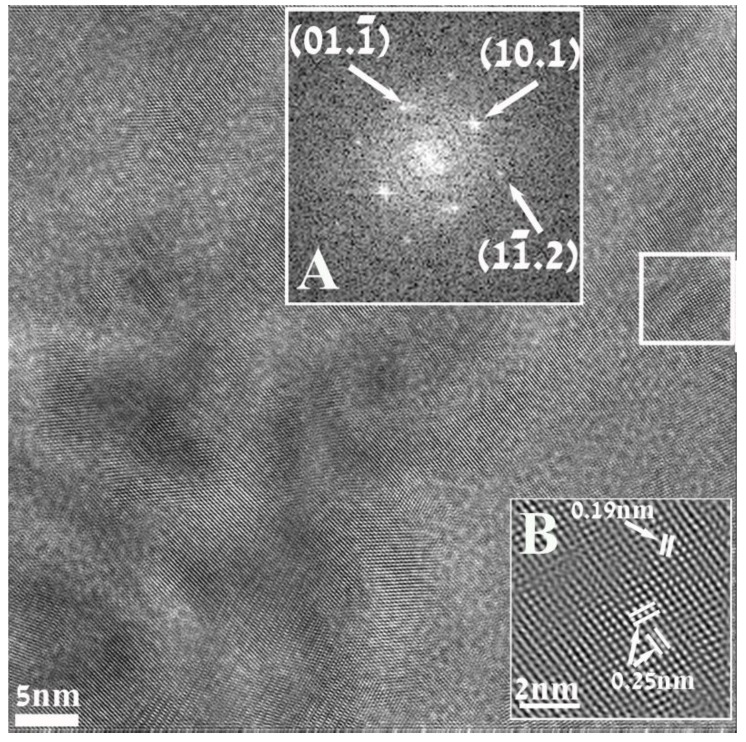
High Resolution (HRTEM) electron micrograph of a nanoparticle resulting in this reaction. Inset (**A**) is the indexed Fourier Transform (FFT) taken from the area marked by the white square. The indexing corresponds to the hexagonal structure describing of ZnO. Inset (**B**) represents the magnified and processed portion of the image outlined by the white square, displaying the interplanar spacing for d_10.1_ and d_10.2_ family of planes.

Figure 3 is a HRTEM image of a nanoparticle of ZnO, displaying well resolved lattice-fringe contrast and the identification was also based on the analysis of such high resolution images. Inset A represents the Fourier transform (FFT) of the portion of the image (white square) and looks like a diffraction pattern, geometrically identical to that which would be recorded in a diffraction experiment. Analysis of this pattern revealed sets of reflections that could be readily referred to the hexagonal structure of (unit cell *a* = 3.25 Å; *c* = 5.21 Å pdf file # 01-0899-1397) and were successfully indexed. Inset B represents the processed and magnified portion of the image outlined by the white square. The distances measured between lattice fringes were 0.25 nm and 0.19 nm, matching the interplanar spacing for *d*_10.1_ and *d*_10.2_ family of planes.

Using two different kinds of electron diffraction techniques (Figure 2 insets A and B) together with FFT analysis of the high resolution images, (Figure 3 inset A) showed that in this synthesis the crystalline hexagonal ZnO is formed.

**Figure 4:**
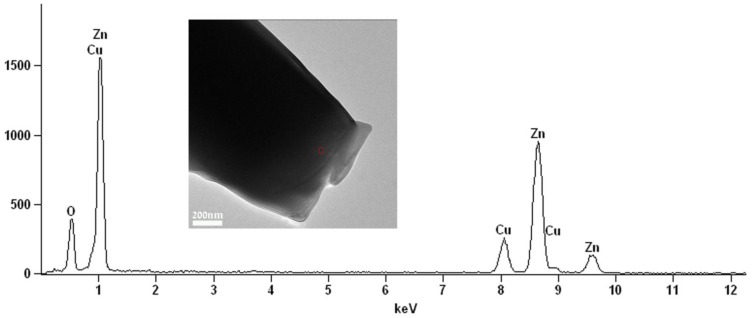
Energy dispersive spectroscopy (EDS) spectrum recorded with a 35 nm electron probe from the “cauliflower-like” ZnO microparticle (marked by the circle on the inset image) showing characteristic peaks of the elements Zn and O. The Cu peak is from the specimen grid.

This result is also in good agreement with energy dispersive spectroscopy (EDS) elemental analysis (Figure 4) which shows that the elements Zn and O are in an atomic ratio of 43% and 57%, respectively. Those values conform (considering the precision limits of the EDS) to the atomic ratio in the compound ZnO. No carbon was detected in the EDS analysis, indicating that there are no residuals of the PDVB polymeric template in the obtained ZnO microparticles.

**Figure 5:**
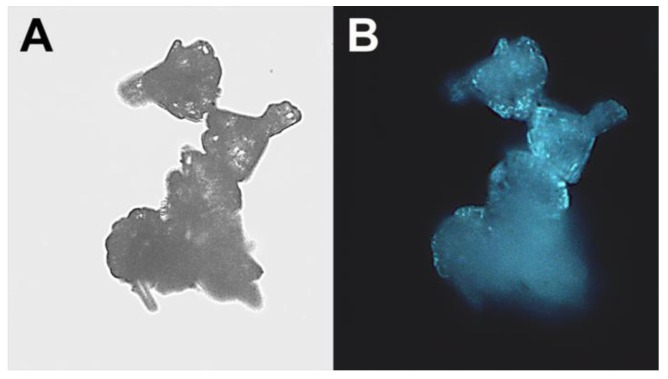
“Cauliflower-like” ZnO microparticles: (**A**) Light and fluorescent; (**B**) microscope images. (Original magnification: ×400).

The “cauliflower-like” ZnO microparticles were further characterized by fluorescence microscopy, and demonstrated fluorescence in the blue regions of the spectrum (Figure 5).

**Figure 6:**
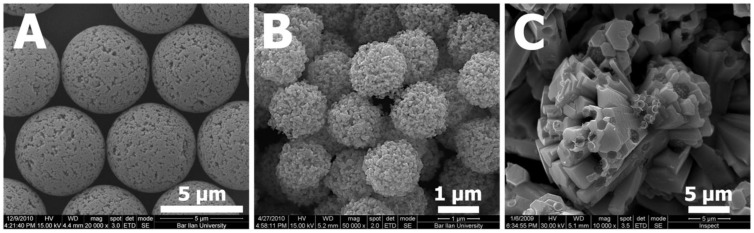
SEM images of (**A**) polydivinyl benzene (PDVB) template microspheres; and (**B**) ZnO microparticles prepared using the zinc acetate precursor; or (**C**) the ZnCl_2_.

In a previous study conducted by our research group, spherical porous ZnO microparticles (Figure 6B) were prepared by a similar procedure to that described above for the formation of “Cauliflower-like” ZnO microparticles (Figure 6C), substituting the ZnCl_2_ precursor by zinc acetate [16].

**Figure 7:**
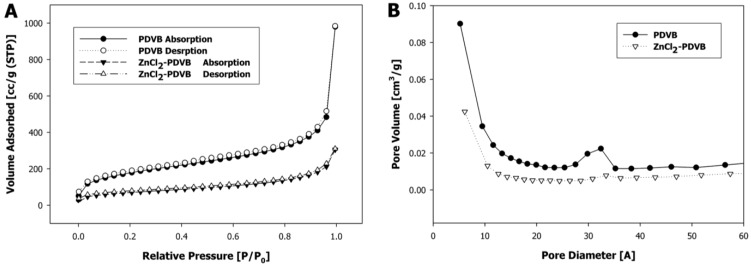
Porous PDVB microspheres before and after ZnCl_2_ insertion: (**A**) N_2_ adsorption-desorption isotherms; (**B**) Barrett–Joyner–Halenda (BJH) pore-size distribution curves.

**Figure 8:**
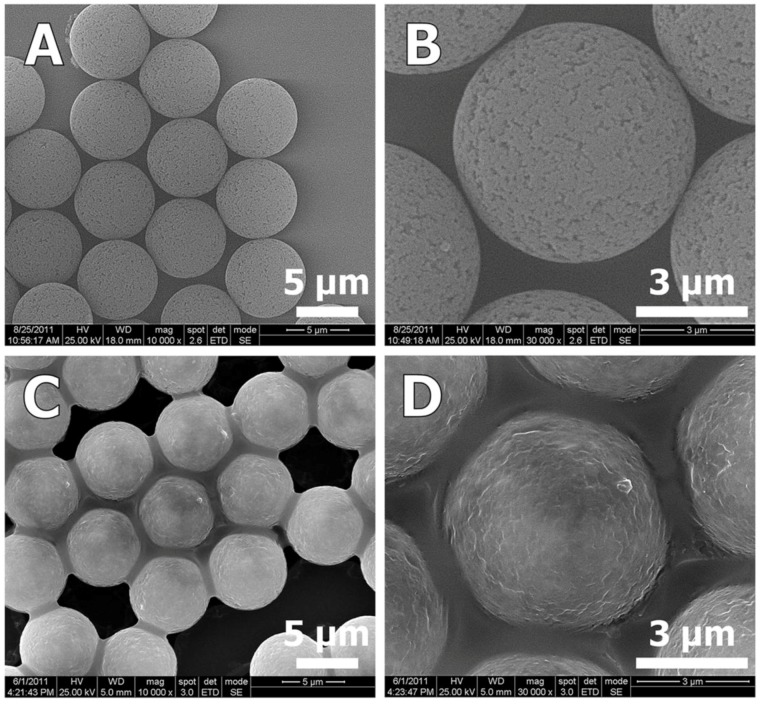
SEM images of PDVB microspheres (**A**,**B**) and ZnCl_2_-PDVB composite microspheres (**C**,**D**). These pictures clearly indicate that the inter-pores of the PDVB microspheres were covered by ZnCl_2_.

In order to understand the formation mechanisms of each of the two different shapes of ZnO microparticles, we examined the Zn distribution within the PDVB template microspheres for both Zn precursors: ZnCl_2_ and zinc acetate. In the first case, the added ZnCl_2_ significantly decreased the surface area of the porous PDVB template microspheres, as calculated by the Brunauer-Emmett-Teller method, from 626 m^2^/g to 245 m^2^/g (Figure 7A). The pore volume, estimated from the desorbed amount of N_2_, was decreased from 1.52 cm^3^/g for the PDVB microspheres to 0.48 cm^3^/g to the ZnCl_2_-PDVB microspheres. In addition, the BJH pore-size distribution curves (Figure 7B) demonstrate that both intra-particle micropores and mesopores volume were decreased by the addition of ZnCl_2_, although the decrease of the mesopores volume is more significant. This suggested that the ZnCl_2_ was penetrated mostly to the inter-particles mesopores, and indeed, SEM images of the ZnCl_2_-PDVB composite microspheres demonstrate clearly that the ZnCl_2_ adsorbed on the PDVB particles’ outer surface (Figure 8).

**Figure 9:**
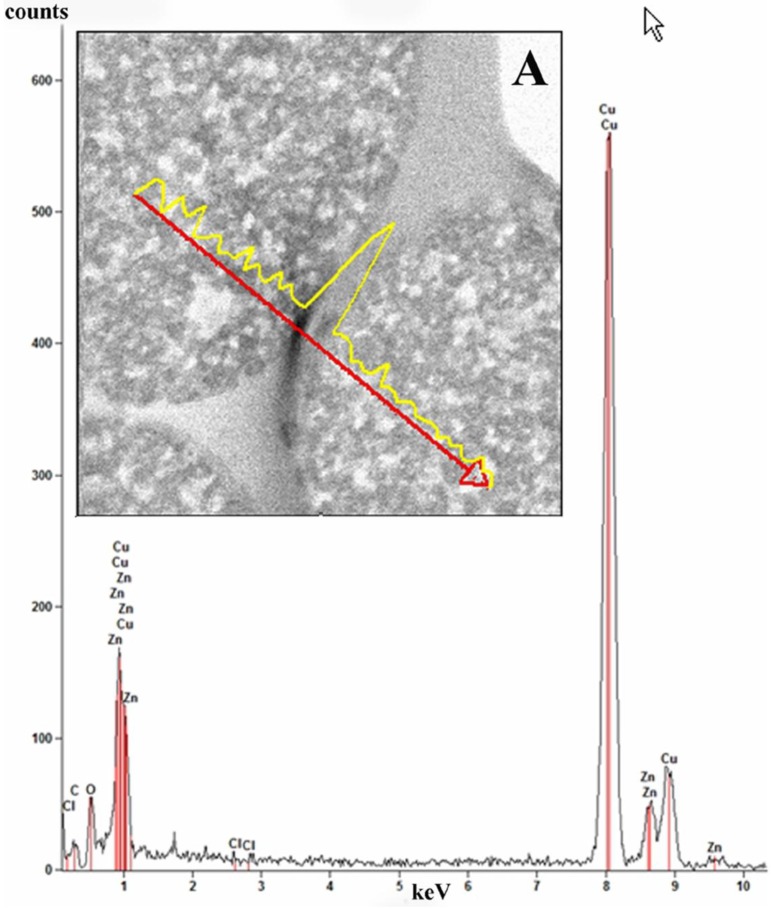
Bright field scanning transmission electron mode (STEM) image of ZnCl_2_-PDVB microspheres. Elemental line scan is shown for Zn (inset **A**). The red arrow marks a line on the surface of the microspheres where the line scan was performed. The figure shows the EDS spectrum recorded from a 35 nm area of a microsphere showing characteristic peaks of the elements C, O, Cl and Zn in the analyzed area. The Cu peak is from the specimen grid.

Elemental line scan and point EDS techniques were used for elemental analysis of the ZnCl_2_ composite microspheres (Figure 9). Elemental analysis was performed in EDS mode and the spectrum (inset B) taken from ~35 nm area of a microsphere shows that the elements Zn, Cl, C, O and Cu (from the specimen grid) are all present in the sample.

Analysis of the distribution of ZnCl_2_ in the ZnCl_2_-PDVB microspheres was conducted in the STEM mode. Figure 9A displays the line scan profile for Zn (yellow) performed on ZnCl_2_-PDVB microspheres.

The line scan performed demonstrates that the ZnCl_2_ nanoparticles are partially embedded in the carbon matrix but are mostly distributed on the surface of the PDVB microspheres.

We suggest that the ZnCl_2_, which was concentrated mostly on the PDVB microspheres surface, was crystallized as ZnO in a typical hexagonal rods structure [19] around the PDVB microspheres. The PDVB organic microspheres were totally combusted during the calcination, leaving porous spherical patterns of ~1–2 μm embossed in the ZnO hexagonal rods and thus providing it “cauliflower-like” appearance. The portion of ZnCl_2_ which was penetrated into the PDVB microspheres, created small round nanoparticles (~10 nm) observed within the spherical deficiencies formed by the PDVB template particles (Figure 6C). ZnCl_2_ is a polar inorganic salt, with low solubility in organic solvents. It is very hygroscopic and even deliquescent, so it might have been dissolved in small amount of water adsorbed to the reaction medium. This aqueous solution does not penetrate to the hydrophobic PDVB microspheres, and therefore, most of the ZnCl_2_ was concentrated outside the PDVB microspheres. We estimate that this small amount of water also interact with the dissolved Zn^2+^ ions to form Zn(OH)_2_, which turn into ZnO by heating [20]:

Zn^2+^ + 2H_2_O → Zn(OH)_2_ + 2H^+^ (1)

**Z**n(OH)_2_ → ZnO + H_2_O (2)

**Figure 10:**
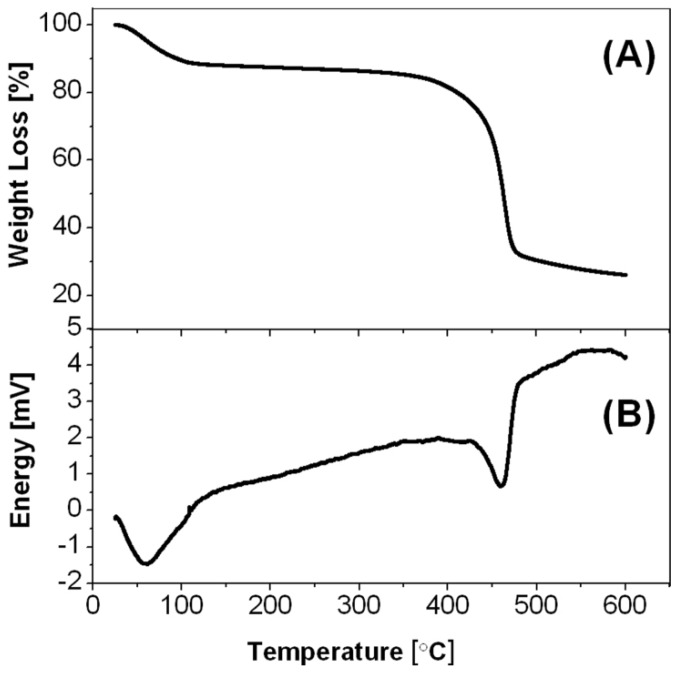
(**A**) Thermogravimetric analysis (TGA); and (**B**) differential scanning calorimetry (DSC) thermograms of the ZnCl_2_-PDVB microspheres.

This estimation is supported by the thermogravimetric analysis of the ZnCl_2_-PDVB microspheres (Figure 10A), demonstrate one slope between 30 and 120 °C, corresponding to the reaction of ZnCl_2_ with water to produce ZnO, and another steep slope between 380 and 540 °C, corresponding to the PDVB template microspheres decomposition, shown also in previous studies [16]. These observations are in good agreement with the DSC results, poses two endothermic peaks: around 60 and around 460 °C (Figure 10B).

In contrast to the ZnCl_2_ precursor, our previous study [16] illustrates that the zinc acetate mostly penetrated into the PDVB microspheres, rather than coating their surface, and therefore decomposed during the calcination to form porous spherical ZnO microparticles, preserving the PDVB template structure shape (Figure 6B). This difference between the two precursors may be explained, at least partially, by the more hydrophobic nature of the zinc acetate relative to ZnCl_2_.

**Figure 11:**
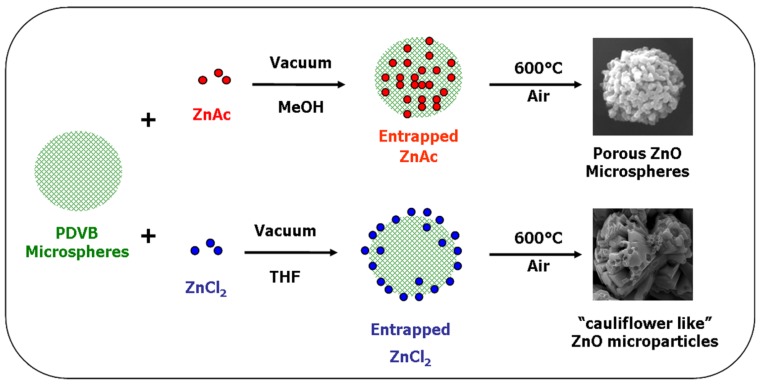
Schematic illustration of the preparation mechanisms of ZnO microparticles with spherical and with “cauliflower-like” shapes from zinc acetate and ZnCl_2_, respectively.

The diverted synthesis mechanisms of the two different structures of ZnO microparticles obtained using zinc acetate and ZnCl_2_, are summarized in Figure 11.

## 4. Summary

The present work describes the preparation of “cauliflower-like” ZnO microparticles, using PDVB polymeric template. This unique structure was formed due to the hydrophilic ZnO precursor that was used, namely: ZnCl_2_. The ZnCl_2_ mostly coated the PDVB template microspheres, penetrating to the inter-particle pores and almost did not penetrated into their intra-pores, in opposed to the precursor zinc acetate which was mostly penetrated into the PDVB microspheres intra-pores, as demonstrated in our previous work. ZnO microparticles of different shapes demonstrate unique optical properties that can be utilized in optics, biosensing, catalysis and medical diagnostics. Further studies of the photo-catalytic performance of those “cauliflower-like” ZnO microparticles are required in order to evaluate the contribution of the microparticles unique structure to the ZnO optical properties.
